# Solar X-ray and Extreme Ultraviolet Imager (X-EUVI) loaded onto China’s Fengyun-3E Satellite

**DOI:** 10.1038/s41377-022-00711-0

**Published:** 2022-02-02

**Authors:** Bo Chen, Guang-Xing Ding, Ling-Ping He

**Affiliations:** Changchun Institute of Optics, Fine Mechanics and Physics, Chinese Academy of Sciences, Changchun, 130033 China

**Keywords:** Imaging and sensing, X-rays

## Abstract

The solar X-ray and Extreme Ultraviolet Imager (X-EUVI), which was developed by CIOMP, is China’s first space-based solar X-ray and Extreme Ultraviolet (EUV) imager; it has been loaded onto the Fengyun-3E Satellite, which is supported by the China Meteorological Administration (CMA), for solar observation. It commenced working on July 11, 2021, and was used to obtain the first X-ray and EUV images in China. X-EUVI employs an innovation dual band design to monitor a much larger temperature range across the Sun, covering the 0.6–8.0 nm wavelength band of the X-ray region and the 19.5 nm band of the EUV region.

*Following the paper published in Light: Science & Applications (*www.nature.com/articles/s41377-019-0157-7*), the same research team recently achieved another first for China*.

The team at Changchun Institute of Optics, Fine Mechanics and Physics, Chinese Academy of Sciences (CIOMP), developed X-EUVI, China’s first space-based solar X-ray and Extreme Ultraviolet (EUV) imager. This imager has been loaded onto the Fengyun-3E Satellite. Launched in July 2021, X-EUVI recorded China’s first batch of solar images in the 19.5 nm and 0.6 nm to 8.0 nm wavelength regions. The images officially released by the CMA on September 2 2021 are shown in Fig. 1. This represents a breakthrough for solar observation in the X-ray and EUV regions in China, and images obtained from the X-EUVI will play an important role in solar research and space weather forecasting^1–5^.

**Fig. 1:**
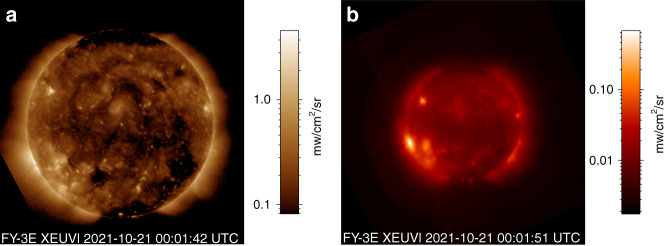
The solar images captured by X-EUVI. **a** image in the 19.5 nm region. **b** image in the 0.6–8.0 nm region

The X-EUVI was developed by the research team in CIOMP. After 5 years of research, the team achieved breakthroughs in four key technologies and independently developed an innovative dual band X-EUVI composed of an X-ray grazing incident optical system and an EUV multilayer normal incident optical system^6,7^. The two systems share a common optical axis and a common CCD detector. The solar images in the X-ray and EUV bands are focused onto a common CCD by an optical switching assembly respectively. The innovative design enables reduced space and weight for space applications.

X-EUVI is also equipped with a solar X-ray sensor and EUV sensor, which works regularly to monitor the absolute solar irradiance and calibrate solar X-ray and EUV images for solar observation. In addition, X-EUVI has autotracking and image stabilization functions, which can be used to track the Sun in real time, rapidly compensate for pointing deviation, and avoid image blurring caused by other device disturbances to obtain high-quality images. The X-EUVI is shown in Fig. 2.

**Fig. 2:**
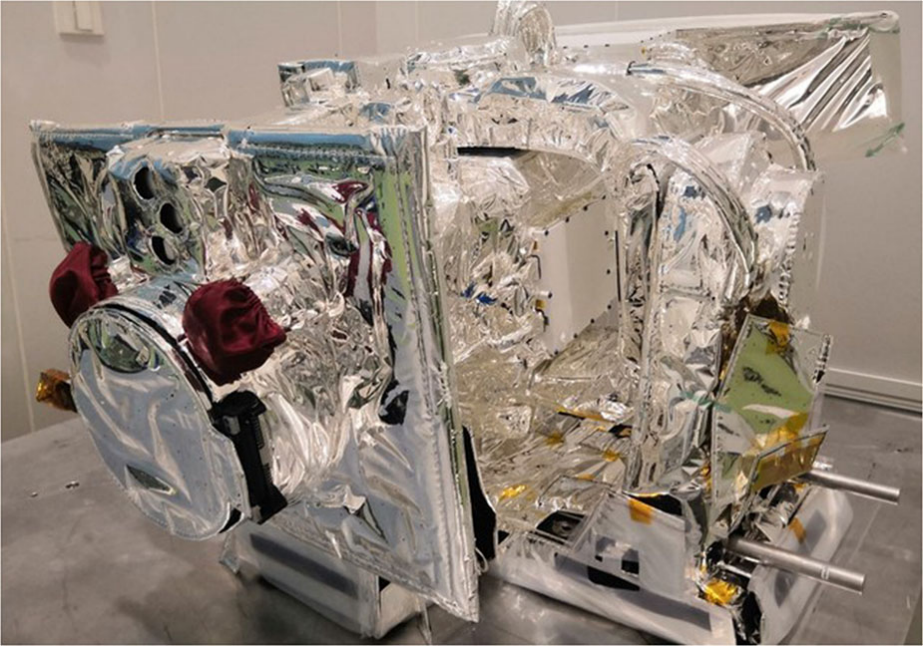
The X-EUVI loaded onto the Fengyun-3E Satellite
